# Sarcoma of the uterine cervix: experience of a single center

**DOI:** 10.1186/s12957-024-03376-8

**Published:** 2024-04-18

**Authors:** Hua Yuan, Lihong Li, Ning Li, Hongwen Yao

**Affiliations:** 1 Department of Gynecologic Oncology, National Cancer Center/National Clinical Research Center for Cancer/Cancer Hospital, Chinese Academy of Medical Sciences and Peking Union Medical College, 17 # Panjiayuannanli, Chaoyang District, Beijing, 100021 China; 2 Department of Pathology, National Cancer Center/National Clinical Research Center for Cancer/Cancer Hospital, Chinese Academy of Medical Sciences and Peking Union Medical College, 17 # Panjiayuannanli, Chaoyang District, Beijing, 100021 China

**Keywords:** Cervical sarcoma, Clinicopathological characteristic, Prognosis

## Abstract

**Objectives:**

To investigate the clinicopathological characteristics and prognosis of patients with primary sarcoma of the uterine cervix.

**Methods:**

We identified all patients with primary cervical sarcomas treated at our institution from 2002 to 2020 and analyzed the clinicopathological characteristics and prognosis.

**Results:**

34 patients were identified, 7 (20.6%) patients had leiomyosarcoma, 6 (17.6%) had carcinosarcoma, 5 (14.7%) had Ewing sarcoma, 4 (11.8%) had rhabdomyosarcoma, 4 (11.8%) had undifferentiated sarcoma, 2 (5.9%) had adenosarcoma, 2 (5.9%) had endometrial stromal sarcoma, 1 (2.9%) had dermatofibrosarcoma protuberans, 1 (2.9%) had alveolar soft tissue sarcoma and 2 (5.9%) had sarcoma not otherwise specified. The median age of the whole patients was 43.5 years (range, 13–63). The median age of patients with Ewing sarcoma or rhabdomyosarcoma was 22 years (range, 13–39) and 17 years (range, 13–36 years), respectively. The distribution by stage was: stage I in 21 (61.8%) patients, stage II in 4 (11.8%), stage III in 6 (17.6%) and stage IV in 3 (8.8%). Overall, 30 patients (88.2%) received surgical treatment. The median follow-up was 33.3 months (range 3.6–187.3 months). 11 patients died within 2 years after diagnosis, most of them were patients with carcinosarcoma or undifferentiated sarcoma (45.5%, 5/11). In the entire cohort, 2- and 5-year OS were 67.2% and 56.9%, respectively. 5-year OS was 25.0% for undifferentiated sarcoma, 50.0% for rhabdomyosarcoma, 50.0% for carcinosarcoma, 53.3% for Ewing sarcoma, 57.1% for leiomyosarcoma.

**Conclusion:**

Cervical sarcomas are rare neoplasms with multiple histological subtypes and follow an aggressive course. Prognosis may be associated with tumor histology and stage.

**Supplementary Information:**

The online version contains supplementary material available at 10.1186/s12957-024-03376-8.

## Introduction

Cervical cancer is the fourth most common female malignancy worldwide and represents a major global health challenge [1]. According to the WHO histological classification of tumours of the uterine cervix, squamous cell carcinomas account for 70–80% of cervical cancers and adenocarcinomas for 20–25% [2]. Cervical sarcomas are ultra-rare neoplasms with multiple histological subtypes and follow an aggressive course. The common subtype of cervical sarcomas includes leiomyosarcoma, carcinosarcoma or malignant mixed Müllerian tumor, Ewing sarcoma, rhabdomyosarcoma, undifferentiated sarcoma, adenosarcoma, and endometrial stromal sarcoma [3]. 

Because of the low incidence and the lack of prospective studies, it is very difficult to reach conclusions as to the best disease management recommendations for cervical sarcoma. Most clinicopathological data are derived from case reports and retrospective studies of a few cases.

We performed a retrospective study to investigate the clinicopathological characteristics and prognosis of patients diagnosed with primary sarcoma of the uterine cervix in our center.

## Patients and methods

### Patients

Following Institutional Review Board approval, we performed a retrospective analysis of all patients diagnosed with primary sarcoma of the uterine cervix from January 1, 2002 to January 1, 2020 who received treatment in the Department of Gynecological Oncology of Cancer Hospital, Chinese Academy of Medical Sciences, National Cancer Center.

Only patients with a diagnosis of cervical sarcoma confirmed by an experienced gynecologic pathologist in our hospital were included. The full medical records of these patients were included in this study. Clinical and pathologic variables, treatment modalities, and outcomes were assessed. Stage was retrospectively assigned using the International Federation of Gynecology and Obstetrics (FIGO) 2018 staging system for cancer of the cervix uteri [4]. 

### Statistical analyses

For the survival analyses, Overall survival (OS) was defined as the time from the date of diagnosis to death for which cervical sarcoma was the primary or underlying cause. Survival was estimated using the Kaplan–Meier product-limit method, and differences were tested for statistical significance using the log-rank test. Two-sided P values less than 0.05 were considered to be statistically significant. All analyses were performed using the SPSS Statistics20.0 software.

## Results

### Patient characteristics

Thirty-four patients were identified. Of these patients, 7 (20.6%) patients had leiomyosarcoma, 6 (17.6%) patients had carcinosarcoma, 5 (14.7%) patients had Ewing sarcoma, 4 (11.8%) patients had rhabdomyosarcoma, 4 (11.8%) patients had undifferentiated sarcoma, 2 (5.9%) patients had adenosarcoma, 2 (5.9%) patients had endometrial stromal sarcoma, 1 (2.9%) patient had alveolar soft tissue sarcoma, 1 (2.9%) patient had dermatofibrosarcoma protuberans, and 2 patients (5.9%) had sarcoma not otherwise specified (Table 1). Immunohistochemistry results for patients with different sarcoma subtypes were listed in Supplementary Table 1. Photographs (10X, 20X) with haematoxylin eosin staining of representative histomorphology from different sarcoma subtypes were showed in Supplementary Fig. 1.

**Table 1:** Clinicopathological characteristics of patient with cervical sarcoma (*n* = 34)

Clinical Characteristics	n	%
Age (years)		
<40	16	47.1
≥40	18	52.9
**Primary symptoms**		
Vaginal bleeding	24	70.6
Physical examination	7	20.6
Vaginal introital mass	2	5.9
Vaginal drainage	1	2.9
**Tumor size (cm)**		
<5	14	41.2
≥5, <10	13	38.2
≥10	7	20.6
**Pathologic Stage (FIGO 2018)**		
I	21	61.8
II	4	11.8
III	6	17.6
IV	3	8.8
**Pathological subtype**		
leiomyosarcoma	7	20.6
carcinosarcoma	6	17.6
Ewing’s sarcoma	5	14.7
rhabdomyosarcoma	4	11.8
undifferentiated sarcoma	4	11.8
sarcoma not otherwise specified	2	5.9
adenosarcoma	2	5.9
endometrial stromal sarcoma	2	5.9
alveolar soft tissue sarcoma	1	2.9
dermatofibrosarcoma protuberans	1	2.9
**Surgical treatment**		
Yes	30	88.2
No	4	11.8
**Neoadjuvant chemotherapy**		
Yes	7	20.6
No	27	79.4
**Surgery type**		
Radical hysterectomy	14	46.7
Hysterectomy	15	50.0
Tumor resection	1	3.3
No surgery	4	
**Cervical stromal invasion**		
Inner 1/3	8	28.6
Middle 1/3	3	10.7
Outer 1/3	17	60.7
NA	6	
**Lymphvascular space invasion**		
Positive	18	62.1
Negative	11	37.9
NA	5	
**Pelvic lymph node**		
Positive	3	21.4
Negative	11	78.6
NA	20	
**Para-aortic lymph node**		
Positive	1	16.7
Negative	5	83.3
NA	28	
**Adjuvant chemotherapy**		
Yes	21	70.0
No	9	30.0
NA	4	
**Adjuvant radiotherapy**		
Yes	4	13.3
No	26	86.7
NA	4	

*Note Abbreviation NA* not assessed

The median age of the whole cohort was 43.5 years (range, 13–63 years). The median age of patients with Ewing sarcoma or rhabdomyosarcoma was 22 years (range, 13–39) and 17 years (range, 13–36), respectively. All patients with Ewing sarcoma or rhabdomyosarcoma or endometrial stromal sarcoma were diagnosed before 40 years. But the median age of patients with carcinosarcoma or leiomyosarcoma was older, which was 54 years (range, 28–61) and 48 years (range, 38–59), respectively. Twenty-four (70.6%) patients presented with vaginal bleeding which was the most common primary symptoms. 7 (20.6%) patients were diagnosed by physical examination.

The proportion of patients with smaller tumor (<5 cm) was 41.2%, compared to 38.2% for 5 to 10 cm, 20.6% for ≥ 10 cm (Table 1). All patients with Ewing sarcoma had lager tumors (≥ 5 cm). The 2018 distribution by stage was: stage I in 21 (61.8%) patients, stage II in 4 (11.8%) patients, stage III in 6 (17.6%) patients and stage IV in 3 (8.8%) patients (Table 1).

## Treatment

Overall, 30 patients (88.2%) received surgical treatment. Other 4 patients (11.8%) with advanced disease received chemotherapy with (*n* = 3) or without (*n* = 1) radiotherapy.

### Surgical treatment

7 (23.3%) patients received neoadjuvant chemotherapy because of larger tumors. The neoadjuvant chemotherapy regimens included: paclitaxel/platinum (3, 42.9%), doxorubicin/ifosfamide (2, 28.6%), paclitaxel/ifosfamide/cisplatin (1, 14.3%). doxorubicin/ifosfamide/cisplatin (1, 14.3%). For patients who underwent surgical treatment, 15 patients (50.0%) underwent total hysterectomy, 14 patients (46.7%) underwent radical hysterectomy, 1 patient (3.3%) underwent primary tumor resection (Table 1). Of these patients, 17 patients (60.7%) had outer third cervical stromal invasion and 18 patients (62.1%) had lymphvascular space invasion.

14 (46.7%) patients underwent pelvic lymphadenectomy, 6 (20.0%) underwent pre-aortic lymphadenectomy, as part of the initial surgical treatment. 21.4% (3/14) and 16.7% (1/6) patients had pelvic or pre-aortic lymph node metastasis, respectively (Table 1).

### Adjuvant therapy

After surgical treatment, 66.7% (20/30) of them received adjuvant chemotherapy. 13.3% (4/30) of them received pelvic external beam radiotherapy (EBRT), and 13.3% (4/30) patients received vaginal brachytherapy.

The adjuvant chemotherapy regimens included: doxorubicin/ifosfamide/cisplatin (5, 25.0%), gemcitabine/docetaxel (2, 10.0%), doxorubicin/ifosfamide (4, 20.0%), paclitaxel/platinum (3, 15.0%), paclitaxel/ifosfamide (3, 15.0%), doxorubicin/cisplatin (1, 5.0%), etoposide/cisplatin (2, 10.0%).

### Survival analysis

**Fig. 1:**
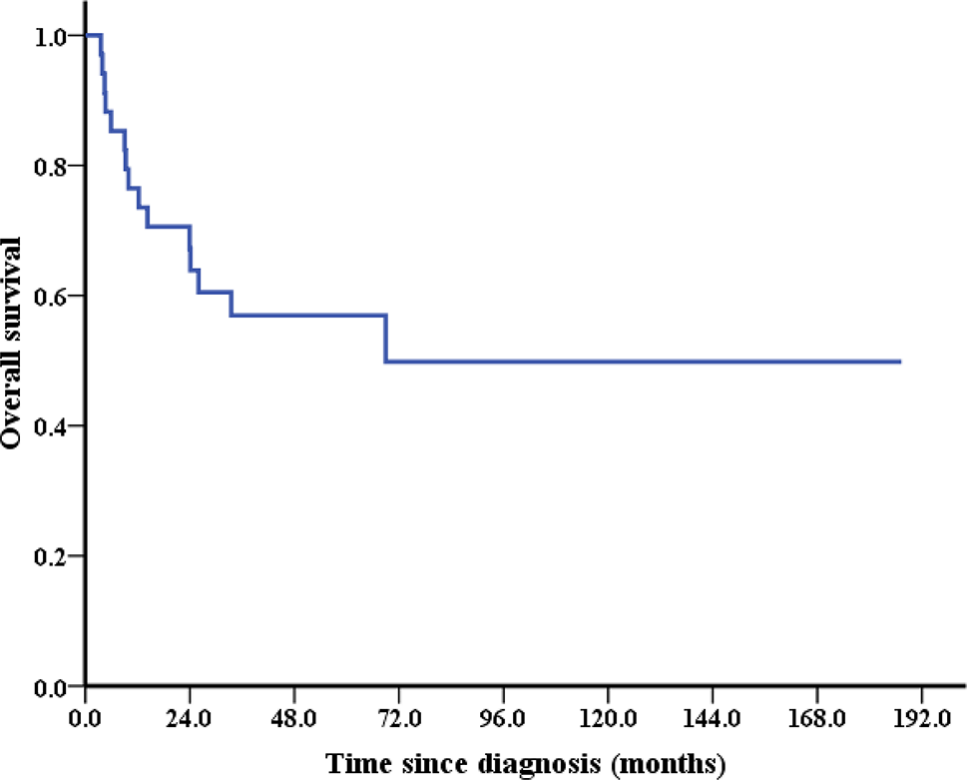
Kaplan–Meier estimates of OS in 34 patients with cervical sarcoma

The median follow-up was 33.3 months (range 3.6–187.3 months). 15 patients died during follow up, and 11 patients died within 2 years after diagnosis, most of them were patients with carcinosarcoma or undifferentiated sarcoma (45.5%, 5/11). 4 patients with advanced stage or metastatic cervical sarcoma received systematic therapy without surgical treatment. All of them died within 1 year after diagnosis. In the entire cohort, 2-year overall survival (OS) and 5-year OS were 67.2% and 56.9%, respectively (Fig. 1).

**Fig. 2:**
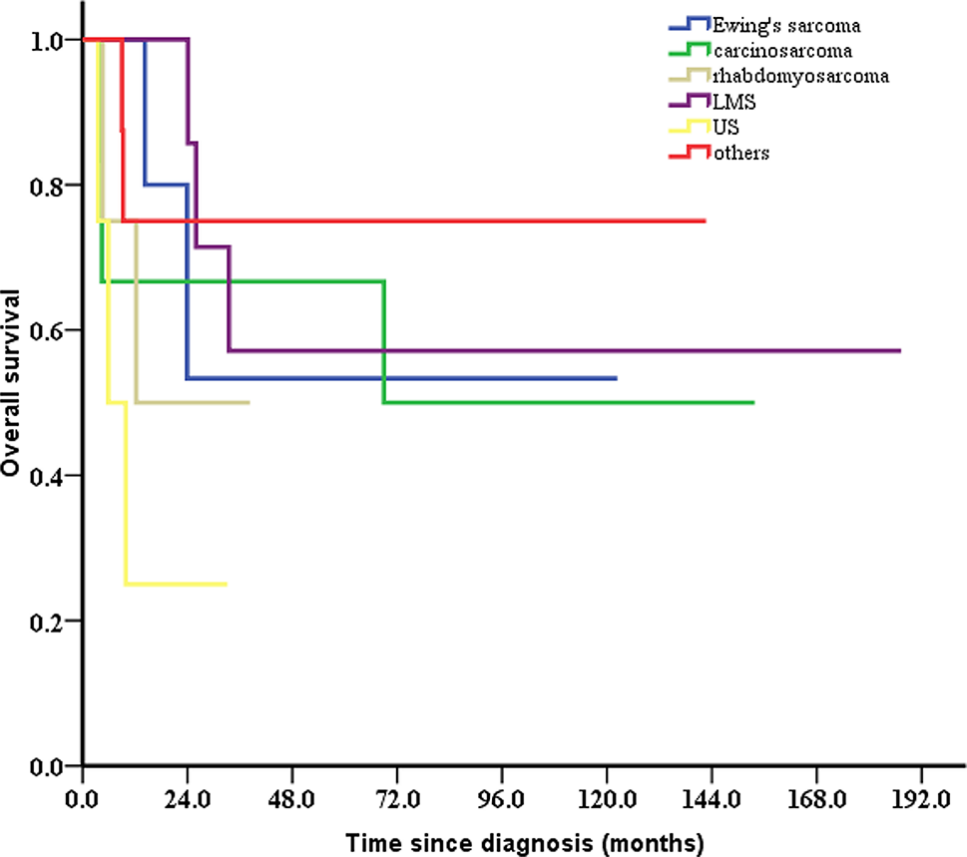
Kaplan–Meier estimates of OS according to the pathological subtype in 34 patients with cervical sarcoma

**Fig. 3:**
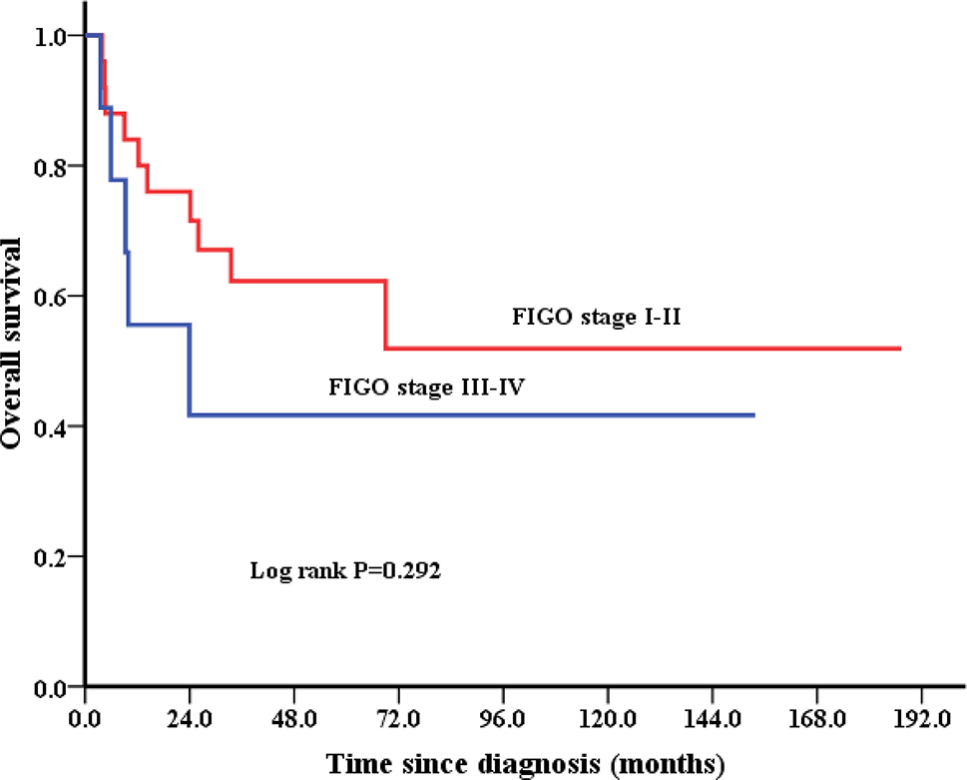
Kaplan–Meier estimates of OS according to the stage in 34 patients with cervical sarcoma

For patients with different pathologic subtype, 5-year OS was 25.0% for undifferentiated sarcoma, 50.0% for rhabdomyosarcoma, 50.0% for carcinosarcoma, 53.3% for Ewing sarcoma, 57.1% for leiomyosarcoma, and 75.0% for others, in the entire cohort (Fig. 2). The 5-year OS for stage I-II and III-IV patients were 62.3% and 41.7%, respectively (Fig. 3). The difference was not significant since the small sample size. For patients who received surgical treatment (*n* = 30), patients with outer third cervical stromal invasion, larger tumor size, and stage III-IV had a non-significantly worse survival (Table 2).

**Table 2:** Kaplan-Meier estimates for the association of tumor factors with OS in patients with cervical sarcoma who received surgical treatment (*n* = 30)

Clinical Characteristics	Cervical sarcoma		5-year OS	P
	n	%		
**Age (years)**				0.845
<40	15	50.0	64.0%	
≥40	15	50.0	64.6%	
**Tumor size (cm)**				0.291
<5	14	46.7	76.0%	
≥ 5	16	53.3	54.1%	
**Pathologic Stage (FIGO 2018)**				0.903
I-II	24	80.0	64.9%	
III-IV	6	20.0	62.5%	
**Neoadjuvant chemotherapy**				0.001
Yes	7	23.3	21.4%	
No	23	76.7	76.6%	
**Surgery type**				0.910
Radical hysterectomy	14	46.7	62.9%	
Hysterectomy or tumor resection	16	53.3	66.5%	
**Cervical stromal invasion**				0.250
Inner 2/3	11	39.3	79.5%	
Outer 1/3	17	60.7	50.5%	
NA	2			
**Lymphvascular space invasion**				0.831
Positive	18	62.1	54.5%	
Negative	11	37.9	59.1%	
NA	1			
**Pelvic/para-aortic lymph node**				0.462
Positive	4	28.6	66.7%	
Negative	10	71.4	60.0%	
NA	16			
**Adjuvant chemotherapy**				0.269
Yes	21	70.0	53.9%	
No	9	30.0	88.9%	
**Adjuvant radiotherapy**				0.838
Yes	4	13.3	75.0%	
No	26	86.7	64.4%	

*NOTE Abbreviation NA* not assessed

## Discussion

Primary sarcomas of the uterine cervix are rare group of tumors with an aggressive behavior and poor outcomes. In the present study, 34 patients with cervical sarcoma treated at our institution from 2001 to 2020 were analyzed. Our series is one of the largest to report on surgical and treatment outcomes for patients with cervical sarcoma in a single center.

Cervical sarcomas are ultra-rare tumors including multiple histological subtypes. Carcinosarcoma is the most common histologic variant and account for 40% of the cervical sarcomas [4]. In our present study, the most common histology of cervical sarcoma was leiomyosarcoma, followed by carcinosarcoma, Ewing sarcoma, rhabdomyosarcoma, undifferentiated sarcoma. The present histology distribution is consistent with previous reported studies.

The age at diagnosis varied according the pathological subtype for cervical sarcoma. Most patients were diagnosed at an early age, especially for patients with Ewing sarcoma or rhabdomyosarcoma or endometrial stromal sarcoma, which were diagnosed before 40 years in our study. Other studies have demonstrated that among cervical cancer patients aged ≤ 25 years, 16 (26.7%) were diagnosed with cervical sarcoma [5]. However, among patients with cervical carcinosarcomas or leiomyosarcoma, the median age at diagnosis was older in the present study. Patients with cervical sarcoma have larger tumors compared with squamous cell carcinomas and adenocarcinomas [6]. We found that more than half of patients had a tumor ≥ 5 cm. Patients diagnosed before 40 years and patients with Ewing sarcoma or rhabdomyosarcoma were more likely to have a large tumor.

Because of their low incidence and the lack of prospective studies, it is very difficult to reach conclusions as to the best disease management recommendations for cervical sarcomas. The treatment of cervical sarcomas is not within the scope of National Comprehensive Cancer Network (NCCN) Guidelines. Multidisciplinary team (MDT) should be recommended for these patients [7]. Cervical sarcoma should be treated with a multimodality management protocol.

Total abdominal hysterectomy/ radical hysterectomy with bilateral salpingo-oophorectomy is the main treatment for patients having disease confined to the cervix. Radical hysterectomy is the important treatment for cervical cancer, but it may not be easily implemented in patients with cervical sarcoma because of the larger tumor size. Emerging evidence suggested that the type of hysterectomy does not have an effect on oncologic outcome in cervical leiomyosarcoma [8]. We found no association between the type of hysterectomy and survival in our cohort. Since most patients with cervical sarcoma are diagnosed at an early age and ovarian involvement is rare, the role of oophorectomy is controversial. For patients diagnosed before 40 years, only 20% of them received oophorectomy in our study.

Neoadjuvant chemotherapy may have impact on down-staging of the tumor to improve the radical curability and safety of surgery; and inhibiting of micrometastasis and distant metastasis [9]. Patients with cervical sarcoma have larger tumors. Neoadjuvant may be a suitable choice for younger patients with a larger tumor. In the present study, 23.3% patients received neoadjuvant chemotherapy before surgical treatment. All these patients achieved a partial response after neoadjuvant chemotherapy and received a surgical treatment. The effect of postoperative treatment among patients who have undergone surgical resection is a subject for debate. Most patients in our cohort received adjuvant chemotherapy, especially for patients with aggressive subtype. Whether the adjuvant chemotherapy should be implemented or not may accord to the pathologic subtype.

The prognosis for women with cervical sarcomas is inferior to that of squamous cell and adenocarcinomas [3, 6]. Cervical sarcomas have an aggressive course. In our study, more than 60% patients had outer third cervical stromal invasion and lymphvascular space invasion. Cervical sarcomas have multiple histological subtypes and the survival rates tend to vary widely according to histological subtypes. We found patients with carcinosarcoma, rhabdomyosarcoma, or undifferentiated sarcoma, had a worsen survival compared with other subtypes, which was in accordance with other studies.

Primary cervical leiomyosarcomas constitute 21% of all cervical sarcomas [8, 10]. Hysterectomy should be the first choice of treatment in these patients according to other studies. Multimodality management may suitable for cervical leiomyosarcoma [11]. Undifferentiated sarcomas are a rare group of tumors with an aggressive behavior and poor outcomes. Survival of cervical undifferentiated sarcoma is extremely poor. We found 3 of 4 patients died within 6 months after diagnosis.

Grayson W et al. firstly report infection with human papillomaviruses (HPV) play an important role in the evolution of cervical carcinosarcoma [12]. Several studies suggest a metaplastic theory of histogenesis of cervical carcinosarcoma [12–15]. In the present study, 33.3% (2/6) patients with cervical carcinosarcoma had HPV infection (HPV type 16 and 55, respectively) and a low-grade squamous intraepithelial lesion. The pathogenesis of cervical carcinosarcoma should be explored in more studies. Furthermore, carcinosarcomas of the uterus are no longer considered sarcomas and may be treated more like a carcinoma [16]. Whether cervical carcinosarcoma should be treated like a carcinoma was unclear.

Ewing sarcomas are small round cell tumors presenting with different degrees of neuroectodermal differentiation. Most patients present with a larger cervical tumor and are diagnosed in children or young adults. Patients with Ewing sarcoma in our study also had a young age onset and a larger tumor. More than half of them had pelvic lymph node metastasis. Rhabdomyosarcomas also tend to occur in children and young women, but appear to have a better prognosis than sarcoma botryoides of the vagina and uterus [17]. Rhabdomyosarcomas comprise 50% of cervical sarcomas in very young women (≤ 25 years) [5]. Many of these started as polypoidal tumours and could be managed surgically. Therapy has recently inclined towards conservative treatment [17]. Adenosarcomas are composed of benign epithelial elements in combination with a malignant stromal component [18]. Patients with adenosarcomas may have a good survival.

There are two limitations to our study. Because of the rarity of cervical sarcoma, the sample size of our study was small. The current study was retrospective, and the primary treatment was not assigned at randomized. Therefore, caution is required when interpreting our results.

## Conclusion

Primary sarcomas of the uterine cervix are rare and aggressive neoplasms. Multimodality therapy is the main treatment for cervical sarcoma. Neoadjuvant chemotherapy may be suitable for these patients. Prognosis vary widely according to histological subtypes.

### Electronic supplementary material

Below is the link to the electronic supplementary material.

Supplementary Material 1

## Data Availability

No datasets were generated or analysed during the current study.

## Abbreviations

FIGO: International Federation of Gynecology and Obstetrics
OS: Overall survival
NCCN: National Comprehensive Cancer Network
MDT: Multidisciplinary team
HPV: Human papillomaviruses
EBRT: External beam radiotherapy
