# Supplementary material for: Sarcoma of the uterine cervix: experience of a single center
Source: World J Surg Oncol. 2024 Apr 18;22:104. doi: 10.1186/s12957-024-03376-8 (PMC11025214; doi:10.1186/s12957-024-03376-8)
Supplement: Supplementary file 1 — Supplementary Material 1 [file 12957_2024_3376_MOESM1_ESM.docx]

**Supplementary Fig. 1** Photographs (10X, 20X) with haematoxylin eosin staining of representative histomorphology from different sarcoma subtypes

1. **Leiomyosarcoma** (10X, 20X)


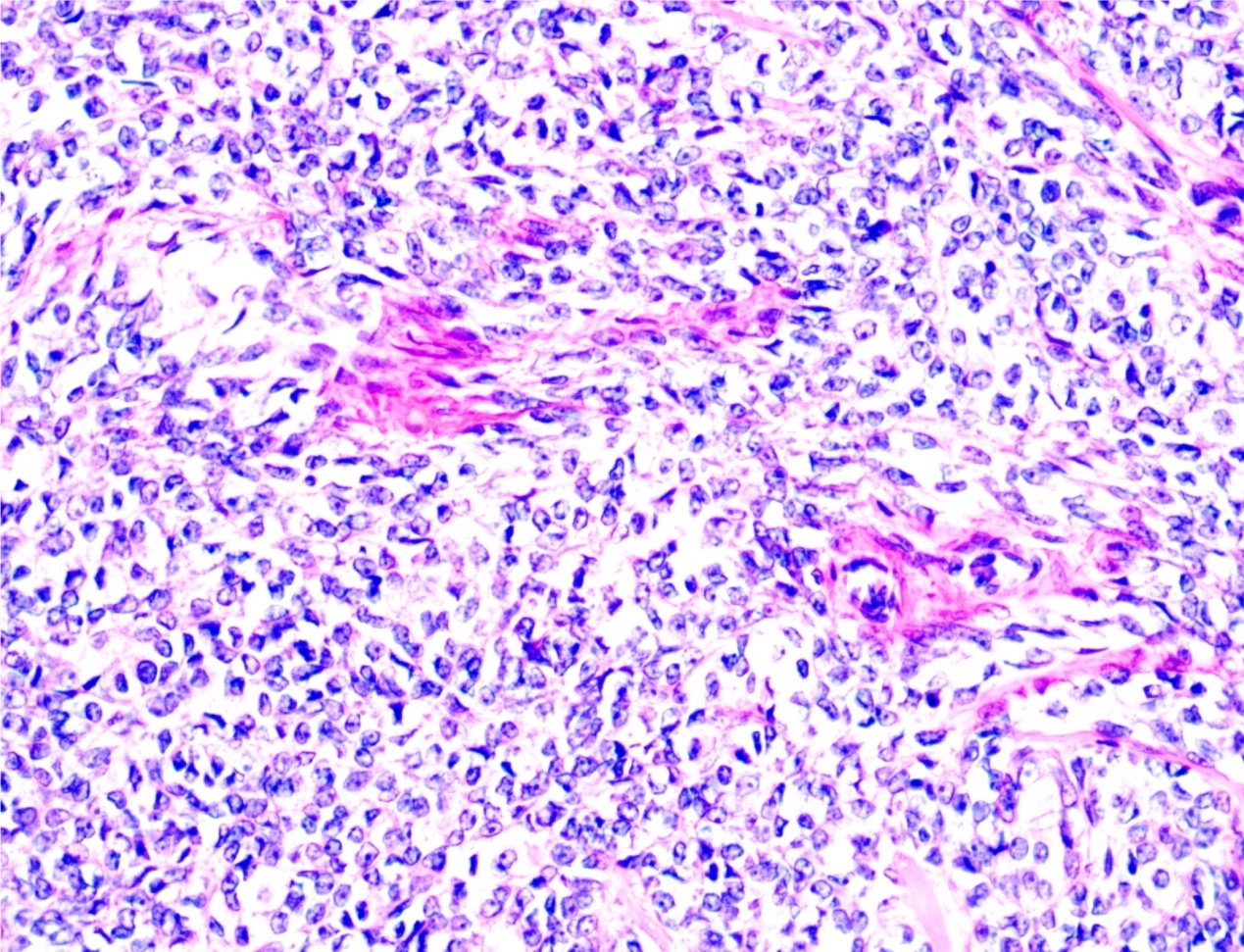


1. **Carcinosarcoma** (10X, 20X)

**malignant epithelial component**


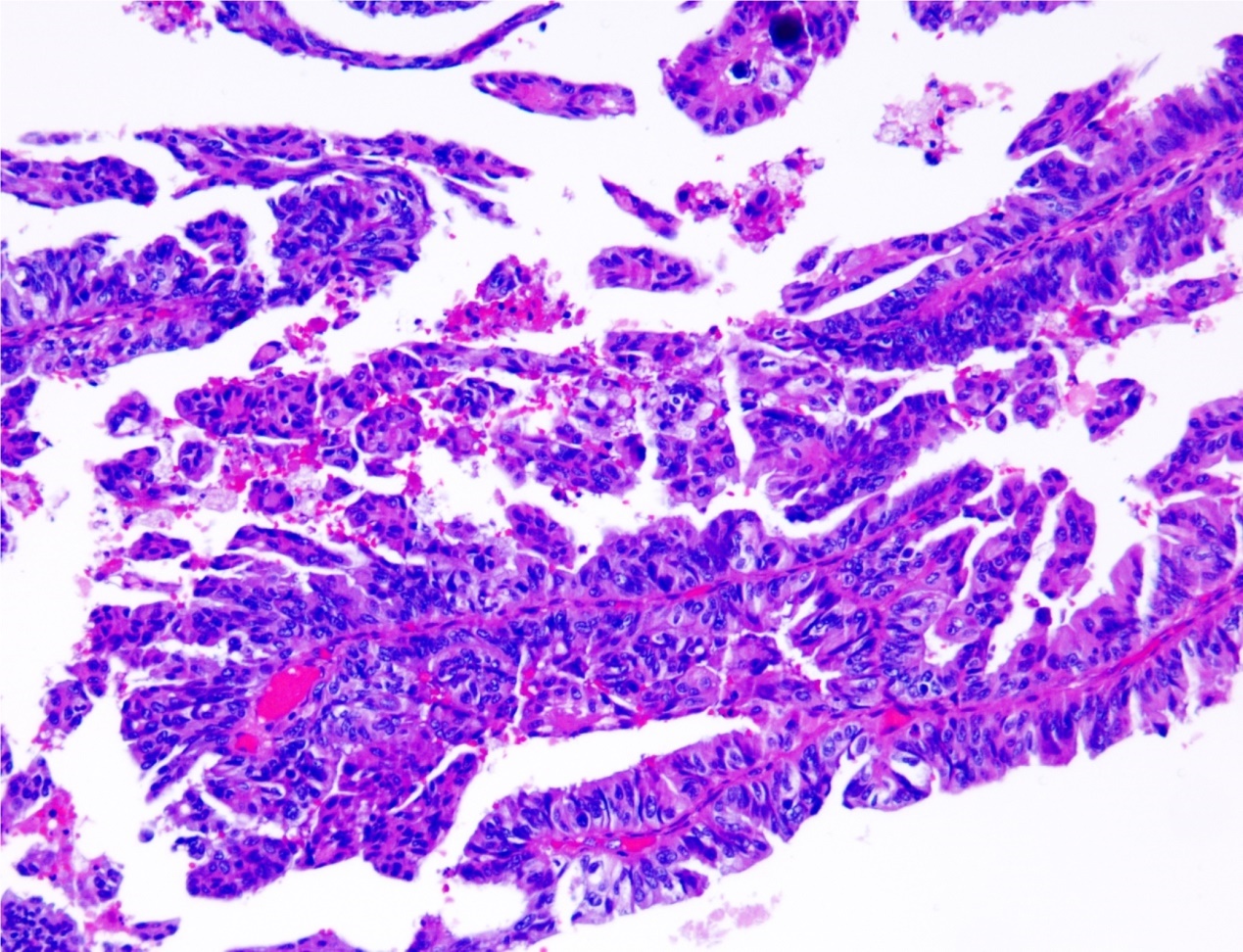


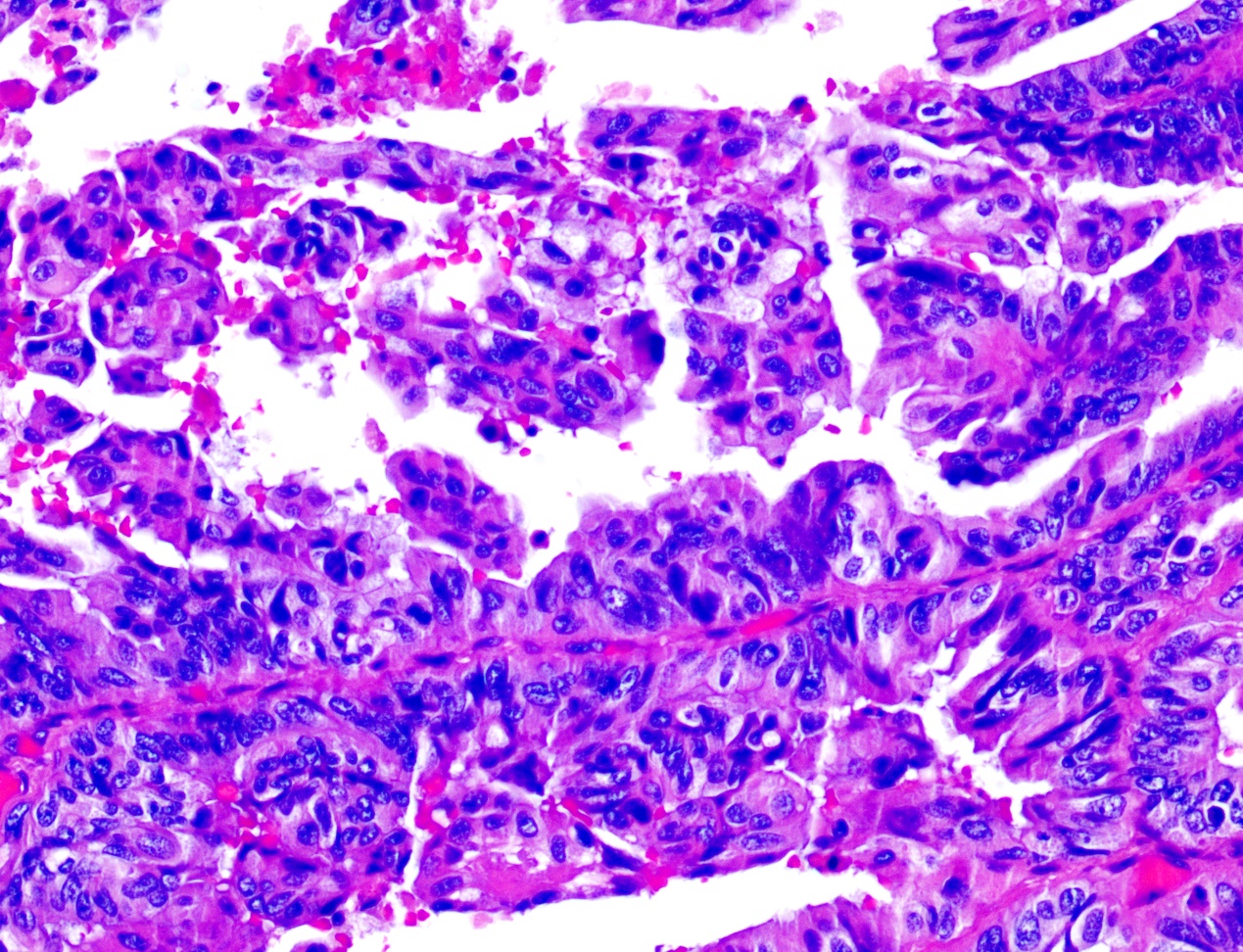


**malignant mesenchymal component**


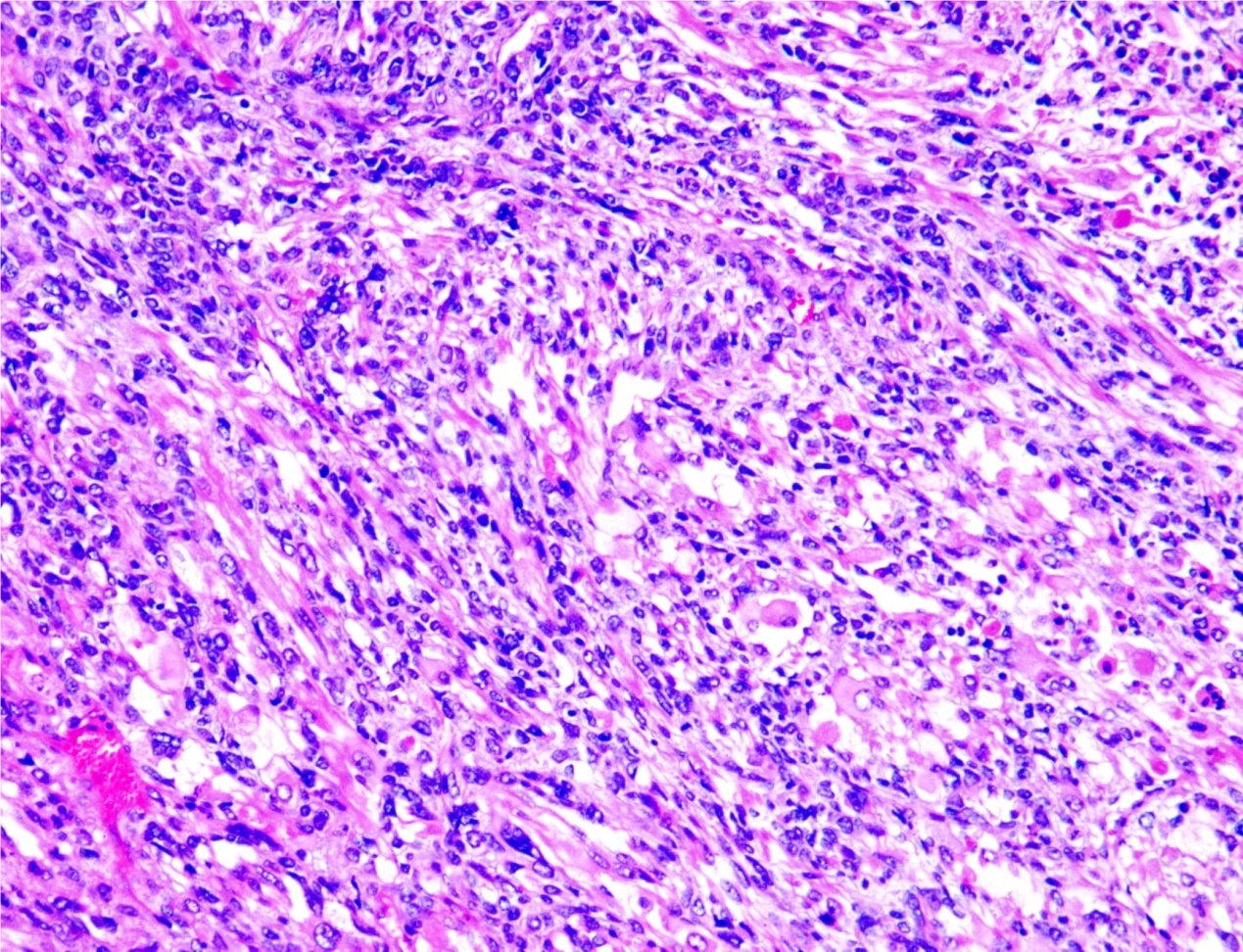


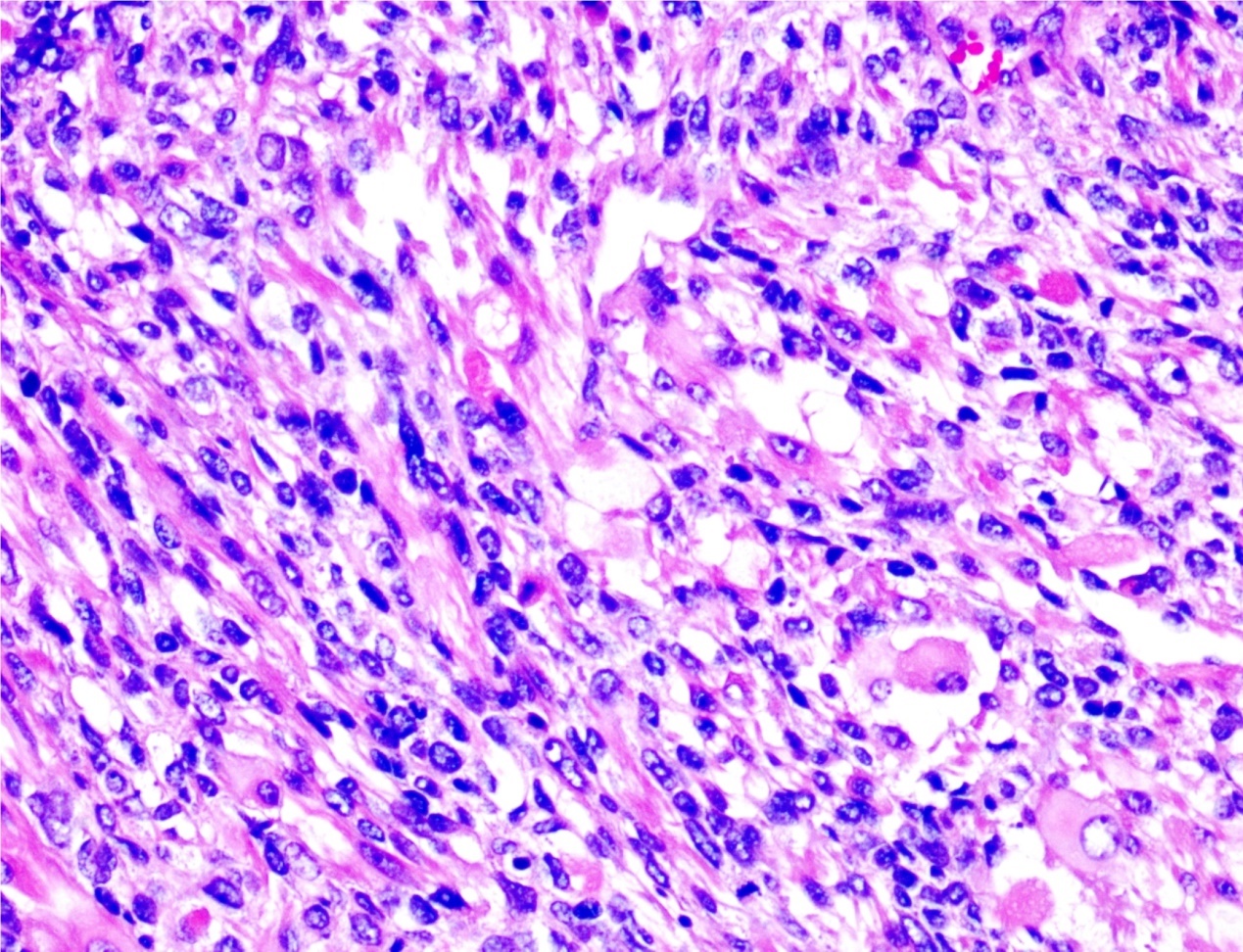


1. **Ewing's sarcoma** (10X, 20X)


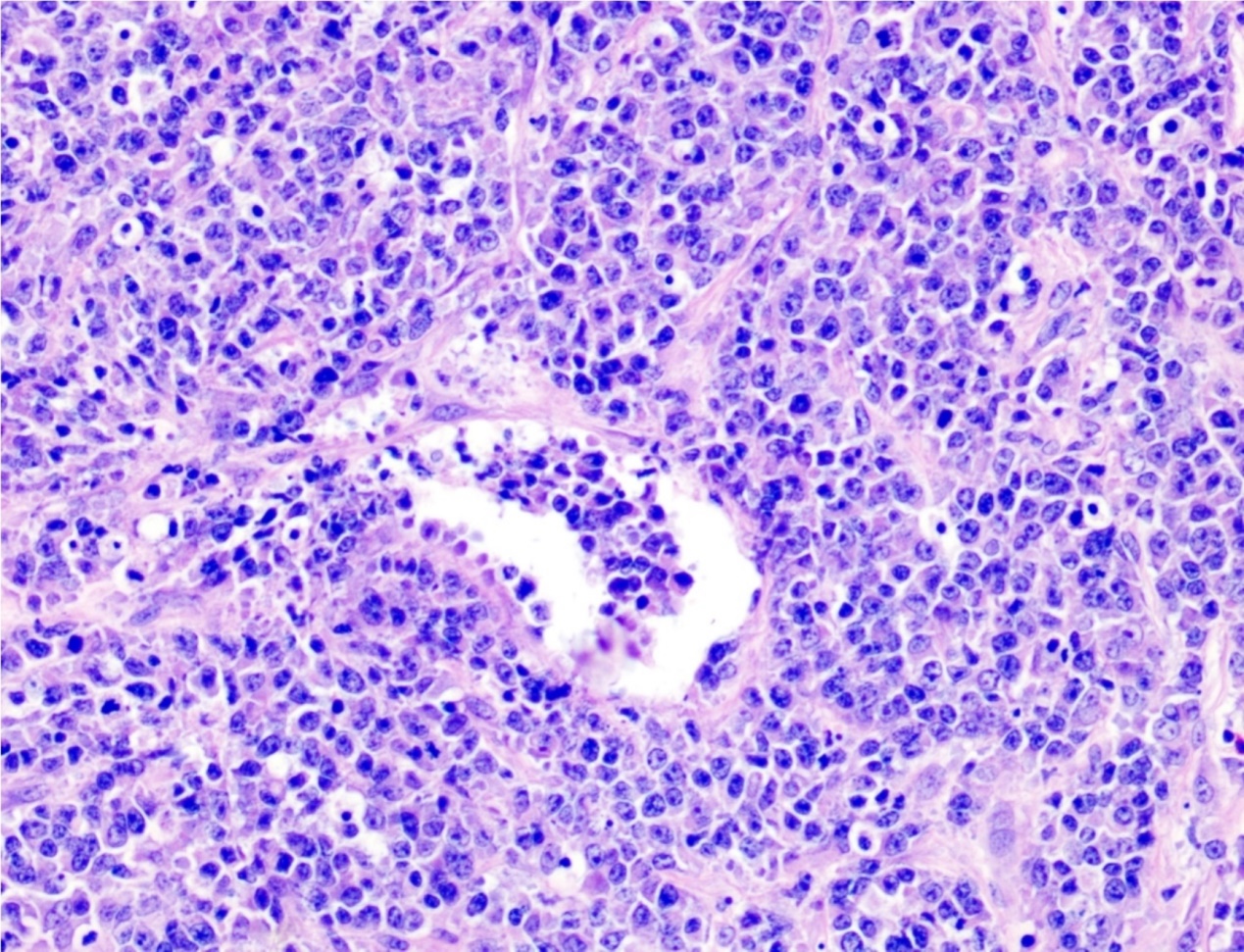


1. **Rhabdomyosarcoma** (10X, 20X)


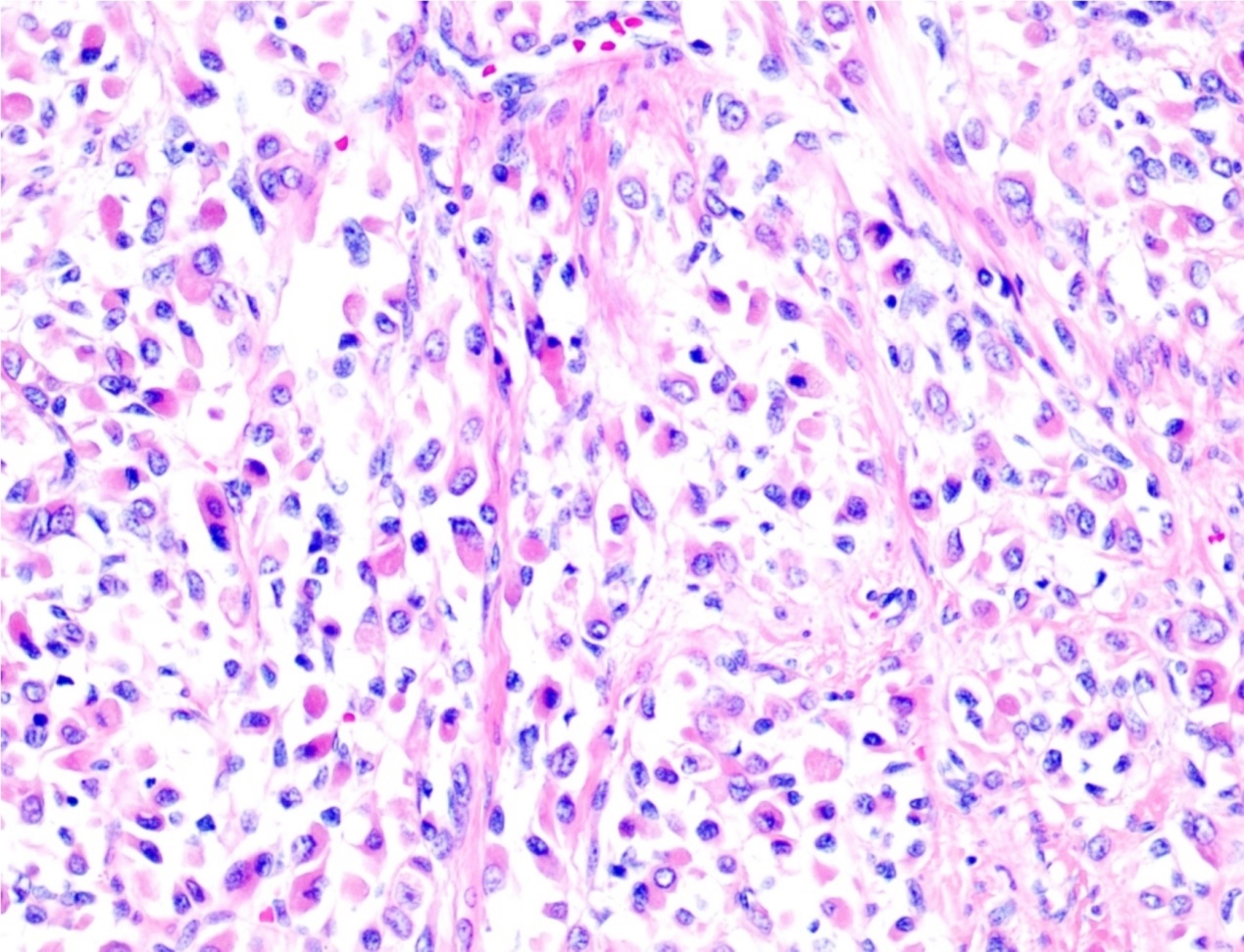


1. **Undifferentiated sarcoma** (10X, 20X)


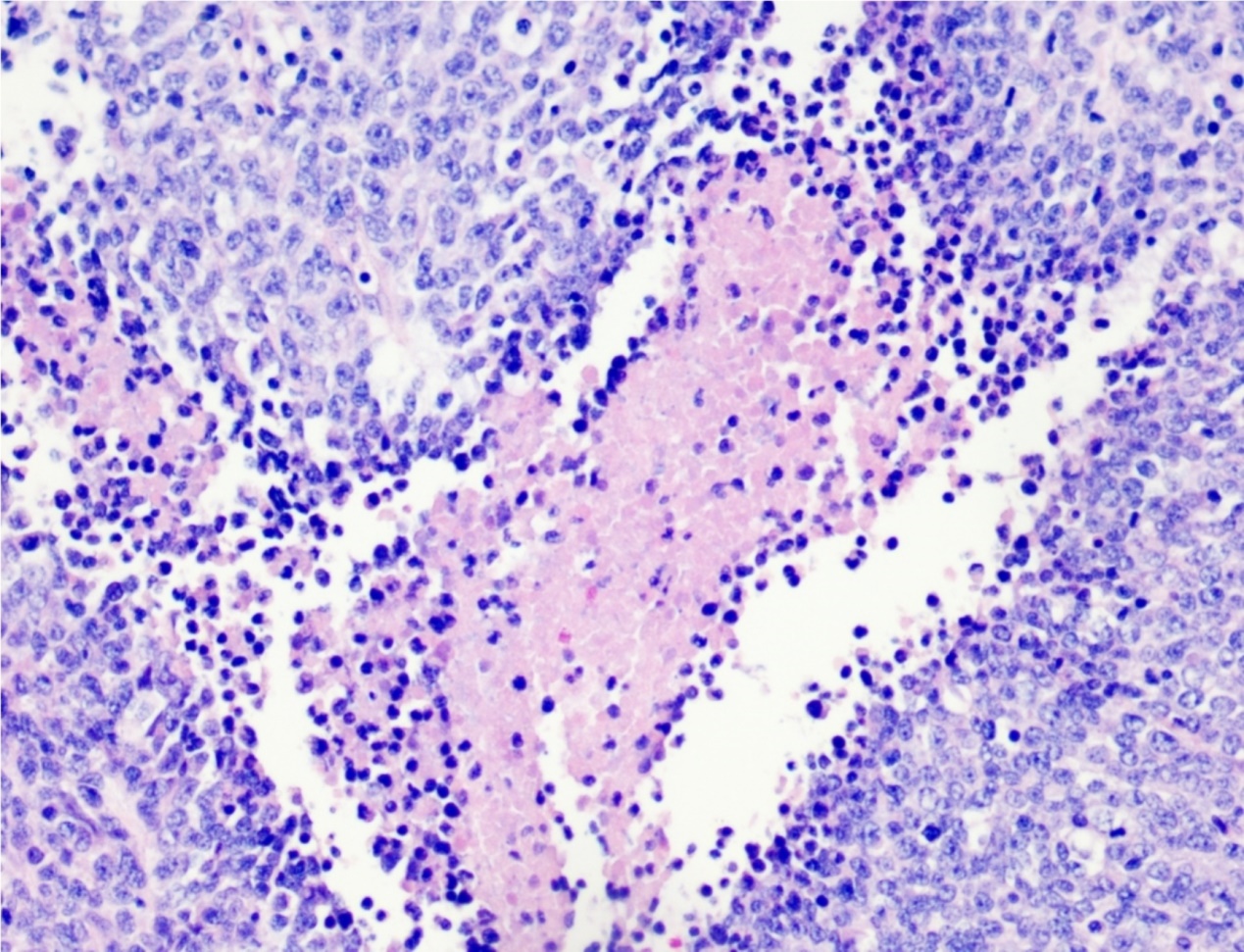


1. **Endometrial stromal sarcoma** (10X, 20X)


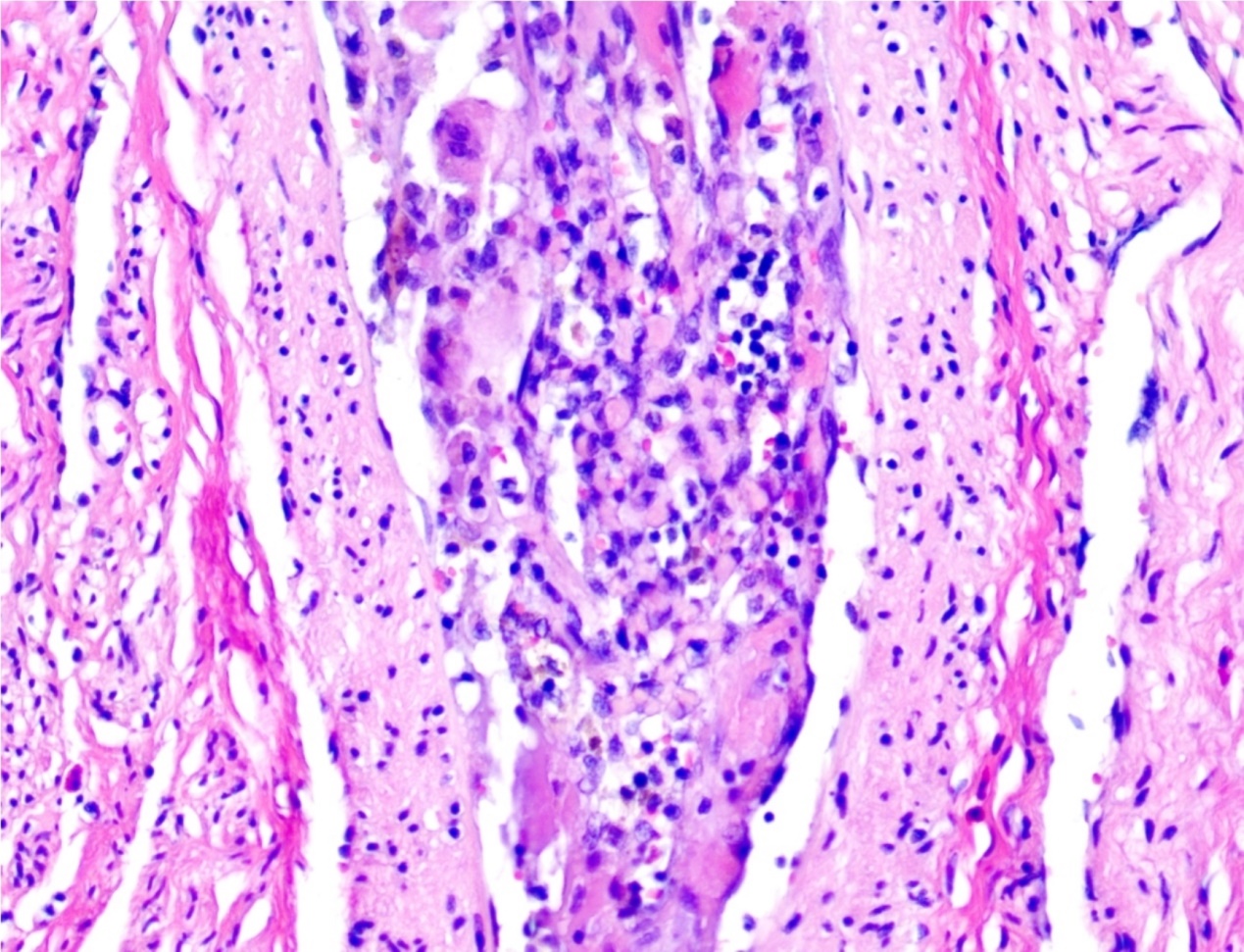


1. **Dermatofibrosarcoma protuberans** (10X, 20X)


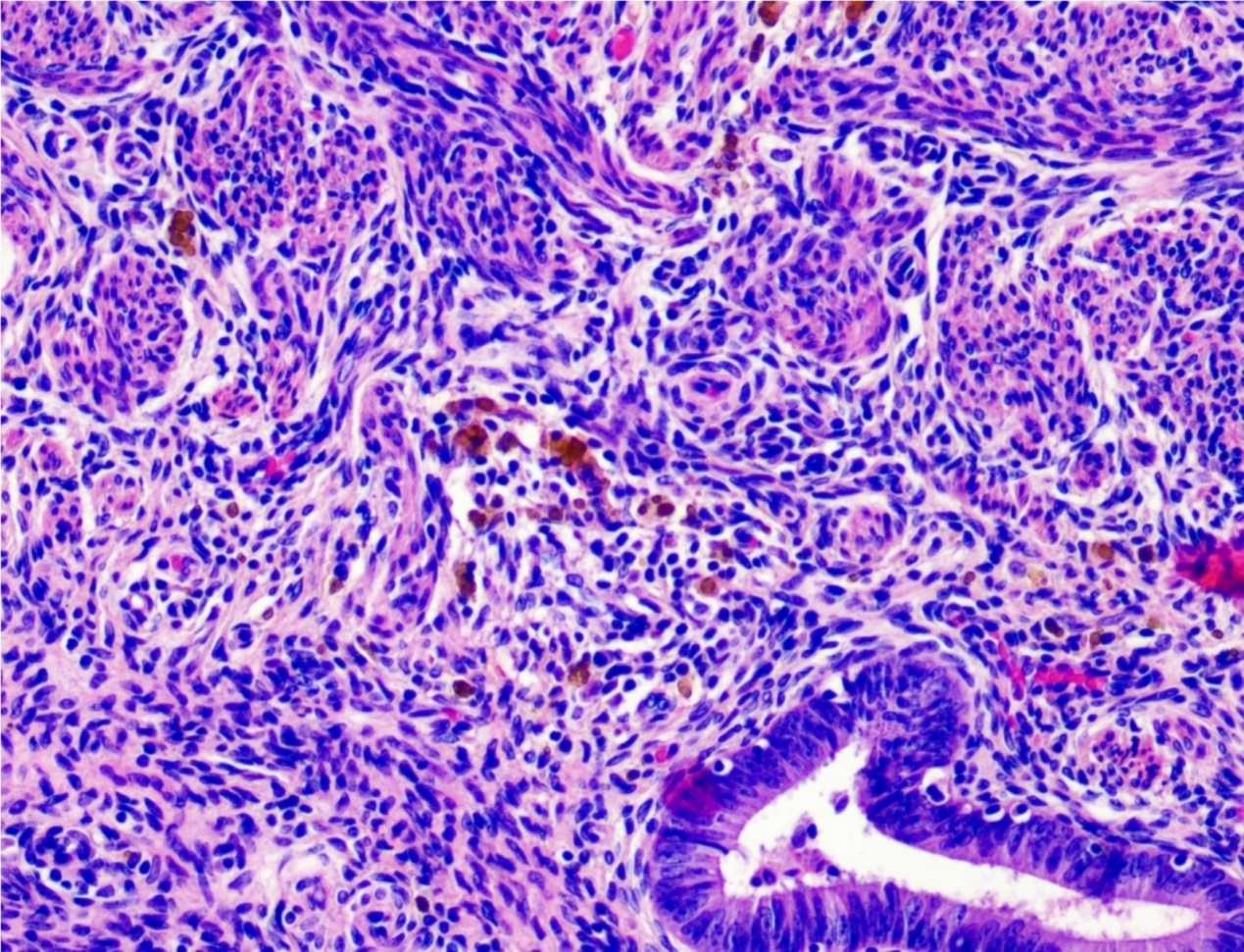


**Supplementary Table 1**. Immunohistochemistry results for patients with sarcoma of the uterine cervix

| **Patient ID** | **Pathological subtype** | **Immunohistochemistry** |
| --- | --- | --- |
| 1 | Ewing's sarcoma | CD99 (3+), NSE (2+), Syn (focal, 1+), CD56 (1+), AE1/AE3 (-), CK18 (-), S-100 (-), ChrA (-), LCA (-), TTF-1 (-), MPO (-), CD43 (-); |
| 2 | Ewing's sarcoma | CD99 (2+), NF (focal, 2+), NSE (3+), Syn (3+), S100 (focal, 3+), GFAP (focal, 2+), AE1/AE3 (epithelioid cells, 2+), CK8 (epithelioid cells, 1+), ChrA (-), CD10 (focal, 1+), SMA (-), α-inhibin (-), AFP (-), HCG (-), Desmin (-), HMB45 (-), LCA (-); |
| 3 | Ewing's sarcoma | CD99 (3+), Fli1 (±), NSE (2+), CD56 (1+), ChrA (-), Syn (-), S100 (1+), MC (-), WT-1 (-), CK5/6 (-), Calretinin (-), AE1/AE3 (-), SMA (1+), Desmin (-), CD10 (-), LCA (-), CD43 (-), MPO (-), Ki-67 (> 20%); |
| 4 | Ewing's sarcoma | CD99 (2+), Fli1 (1+), Syn (1+), CD56 (3+), Sall4 (1+), PAX2 (1+), NF (focal,+), CK8 (1+), AE1/AE3 (-), CK18 (-), EMA (-), P63 (-), P40 (-), CK5/6 (-), ChrA (-), Desmin (-), SMA (-), MyoD1 (-), Myogenin (-), GFAP (-), OCT3/4 (-), PAX8 (-), WT1 (-), LCA (-), Ki67 (+ 80%); |
| 5 | Ewing's sarcoma | CD99 (3+), CD56 (-), CD57 (-), ChrA (-), Fli1 (3+), Ki-67 (+, 40%), NSE (-), S-100 (-), Syn (2+), Vimentin (2+), AE1/AE3 (-), CK20 (-), CK18 (-), EMA (1+), Desmin (-), MyoD1 (-), CD38 (-), CD43 (-), CD10 (1+), MPO (-), TdT (-), CD15 (-), INI1 (3+); |
|  |  |  |
| 6 | carcinosarcoma | AE1/AE3 (3+), CK18 (3+), CK7 (2+), CEA (1+), CA125 (1+), Vimentin (2+), Desmin (-), α-inhibin (-), CD10 (focal, 1+), SMA (1+); |
| 7 | carcinosarcoma | AE1/AE3 (3+), Vimentin (1+), CK8 (1+), CK7 (1+), CK5/6 (focal, 1+), Actin (1+), SMA (focal, 1+), CD117 (-), CD34 (-); |
| 8 | carcinosarcoma | AE1/AE3 (1+), CK18 (1+), CK7 (2+), P16 (3+), CEA (-), CD56 (2+), ER (-), PR (-), S100 (-), EMA (1+), Vimentin (1+), Desmin (-), CD68 (-), Actin (-); |
| 9 | carcinosarcoma | AE1/AE3 (1+), CK18 (3+), CK7 (1+), P16 (3+), CEA (-), CD56 (2+), ER (-), PR (-), S100 (-), Desmin (-), MyoD1 (-), Myogenin (-), Syn (-), ChrA (-), Ki67 (+,50–80%); |
| 10 | carcinosarcoma | AE1/AE3 (epithelioid cells, 3+), CK5/6 (epithelioid cells, focal, 1+), P16 (epithelioid cells, 2+), P63 (-), CEA (-), ER (-), PR (-), CD10 (2+), SMA (-), Desmin (-), Ki-67 (+, 30%); |
| 11 | carcinosarcoma | AE1/AE3 (2+), CK18 (3+), EMA (2+), Vimentin (3+), CK5&6 (focal, 1+), P63 (1+), BCL2 (3+), CD56 (1+), CD99 (2+), Desmin (-), SMA (-), Ki-67 (20%), CD34 (-); |
|  |  |  |
| 12 | rhabdomyosarcoma | AE1/AE3 (1+), Desmin (2+), SMA (focal, 1+), S100 (1+), CK18 (focal, 1+), Myoglobin (2+), CD56 (3+), CK7 (-), MyoD1 (-), Myogenin (-), Vimentin (-), NSE (-), ChrA (-), Syn (-), LCA (-), Ki67 (+ 10%); |
| 13 | rhabdomyosarcoma | AE1/AE3 (-), CD56 (1+), SMA (-), Desmin (1+), Ki-67 (+, 60%), S-100 (-), MyoD1 (1+), Myogenin (1+); |
| 14 | rhabdomyosarcoma | AE1/AE3 (-), CD10 (-), SMA (-), Desmin (focal, 1+), Ki-67 (hotspot, 1+, 70%), MyoD1 (focal, 1+), Myogenin (focal, 1+); |
| 15 | rhabdomyosarcoma | AE1/AE3 (-), CD10 (-), SMA (-), Desmin (focal, 1+), Ki-67 (1+, 40%), MyoD1 (focal, 1+), Myogenin (2+), Actin (3+); |
|  |  |  |
| 16 | leiomyosarcoma | Desmin (2+), AE1/AE3 (-), CD10 (-), Vimentin (1+), Actin (3+), CD99+, α-inhibin (-), ER (focal, 2+), PR (2+), HMB45 (-), EMA (-); |
| 17 | leiomyosarcoma | Desmin (3+), CK18 (1+), AE1/AE3 (-), SMA (1+), Caldesmon (3+), Calponin (3+), S-100 (-), DOG1 (±), CD34 (-), CD117 (-), P16 (-), P53 (-), Ki-67 (+, 20%); |
| 18 | leiomyosarcoma | Desmin (1+), SMA (3+), Actin (2+), MyoD1 (focal, 1+), Myoglobin (-), CD10 (-), α-inhibin (-), AFP (-), HMB45 (-), Melanoma-pan (±), S100 (-), Ki-67 (focal, 1+, 1–2%), PAS (focal, 1+）; |
| 19 | leiomyosarcoma | Desmin (2+), CK (-), Vimentin (2+), SMA (2+), MSA (1+), calponin (1+), s-100 (-), CD34 (-), CD117 (-), ki-67 (40%+); |
| 20 | leiomyosarcoma | Desmin (-), MyoD1 (-), CD31 (-), CD34 (-), MDM2 (-), CD68+, Ki-67 > 30%, AE1/AE3 (±), CD10 (2+), Caldesmon (3+), Calponin (1+), ER (-), PR (1+), Vimentin (3+), ki-67 (+, 60%); |
| 21 | leiomyosarcoma | Desmin (-), Bcl-2 (3+), Caldesmon (2+), Calponin (-), CD10 (-), CD117 (-), CyclinD1 (-), Ki-67 (+, 10%), SMA (-), WT-1 (1+), CD34 (-), S-100 (-), MDM2 (1+), ALK (-); |
| 22 | leiomyosarcoma | Desmin (3+), CD10 (±), CR (-), Caldesmon (2+), D2-40 (-), EMA (focal, 1+), ER (2+), MC (-), PR (2+), SMA (2+), WT1 (-); |
|  |  |  |
| 23 | undifferentiated sarcoma | Vimentin (3+), EMA (focal, 1+), CD10 (focal, 1+), AE1/AE3 (focal, 1+), CyclinD1 (2+), Desmin (-), Caldesmon (2+), ER (-), PR (-), P63 (-), P40 (-), Ki-67 (+, 70%), LCA (-), WT-1 (-), P16 (focal, 1+), P53 (+, 25%); |
| 24 | undifferentiated sarcoma | Vimentin (3+), CD99 (3+), CD117 (2+), CD10 (-), Desmin (-), SMA (-), ER (-), PR (-), AE1/AE3 (-), DOG1 (-), CD34 (-), Ki-67 (+, 70%), CK5/6 (-), CR (-), MC (-), D2-40 (-); |
| 25 | undifferentiated sarcoma | Vimentin (3+), WT (±), CD10 (3+), ChrA (-), ER (-), PR (1+), AE1/AE3 (-), CK18 (-), CK7 (-), Melanoma-pan (-), SMA (-), Syn (-), CD30 (-); |
| 26 | undifferentiated sarcoma | Vimentin (3+), CK5/6 (-), P16 (1+), P40 (-), P63 (-), AE1/AE3 (-), CK18 (-), CD10 (-), Caldesmon (3+), Cyclin D1 (2+), Desmin (-), SMA (-), CD34 (-), S100 (focal, 1+), HMB45 (-), Ki67 (+, 80%); |
|  |  |  |
| 27 | adenosarcoma | Vimentin (3+), WT1 (2+), CD10 (2+), P53 (2+), CEA (-), P16 (-), PR (2+), ER (+), Calponin (-), Desmin (-), SMA (-), Ki-67 (+ 30%); |
| 28 | adenosarcoma | Vimentin (3+), WT1 (2+), CD10 (3+), P53 (3+), CEA (-), P16 (3+), PR (1+), ER (-), Calponin (-), Desmin (-), SMA (-), Ki-67 (+, 70%); |
|  |  |  |
| 29 | endometrial stromal sarcoma | AE1/AE3 (-), Actin (-), CD10 (-), CD30 (1+), CD34 (-), CD49 (-), CD117 (-), Desmin (-), Vimentin (2+), EMA (-), HMB45 (-), MyoD1 (-), Myogenin (-), S100 (-), SMA (-), Ki67 (28%); |
| 30 | endometrial stromal sarcoma | AE1/AE3 (-), Actin (-), CD10 (3+), CD30 (±), CD34 (-), Desmin (-), Vimentin (1+), EMA (-), HMB45 (-), MyoD1 (-), Myogenin (-), S100 (-), SMA (-), CD99 (1+); |
|  |  |  |
| 31 | alveolar soft tissue sarcoma | CgA (-), Syn (-), SMA (-), Desmin (-), CD10 (-), S-100 (-), AE1/AE3 (-), Vimentin (-), ER (-), PR (-), PAS (1+), HMB45 (-); |
|  |  |  |
| 32 | dermatofibrosarcoma protuberans | CD34 (2+), CD10 (-), WT1 (membrane, 1+), Desmin (-), Caldesmon (2+), SMA (-), Calponin (-), CyclinD1 (-), CD117 (-), BCL2 (-), Ki-67 (+, 40%), EMA (-); |

*Note* Immunohistochemistry results for patients with sarcoma not otherwise specified were not listed in the table
